# The Care of Horses

**Published:** 1885-10

**Authors:** 


### The Care of Horses.

The Hon. J. E. Russell, secretary of the Massachusetts board of agriculture, has been lecturing in Worcester on the horse. Here are some of the points he makes:

The fact that the horse has no power to store bile indicates the great rapidity of his digestion and power of assimilation. For such reasons he should be fed as frequently as convenient, and I consider, if a rule can be applied, that a horse should be allowed not more than two per cent. of his weight a day in food; that is, a horse weighing 1,000 pounds should have 20 pounds of food a day, half of which, when at hard work, may be grain. This is an abundant allowance, and in idle time should be reduced at least a quarter. He should have what salt he requires, himself to be the judge.

While I never would turn him to pasture, he should have some green food in summer and carrots in winter. Indian corn, whole or in meal, is unfit food for horses; it is heating and fattening; no horseman wishes to see a fat horse; fatten steers, sheep or hogs, but not horses or men. Oats are the best grain for horses, and the cheapest in the end; if we had some means of crushing or bruising them they would be worth twenty-five per cent. more to us. If I was a miller I would put in a set of rolls and crush oats, and in six months I would have all the business of my region. No man that has ever used crushed oats will have any other.

The best time to water a horse is an hour before or an hour and a half after eating. Suppose his master takes him to the watering trough immediately after eating and his stomach is full of food and he drinks a pail or two pails of water? The consequence is, a portion of the food is forced out of the stomach and is swept along into the larger intestines without assimilation. In France, I saw some horses fed coarse beans, immediately after allowed to drink all the water they would, and then killed and dissected, and some of the beans were found twenty-six feet distant from the stomach, in the intestines.

